# Vaccination protects against acute respiratory distress syndrome (ARDS) in hospitalized patients with COVID-19

**DOI:** 10.1007/s10238-023-01293-w

**Published:** 2024-01-27

**Authors:** Julian Madrid, Prerana Agarwal, Katharina Müller-Peltzer, Marvin Askani, Leo Benning, Mischa Selig, Philipp Diehl, Johannes Kalbhenn, Georg Trummer, Stefan Utzolino, Tobias Wengenmayer, Hans-Jörg Busch, Daiana Stolz, Siegbert Rieg, Marcus Panning, Christopher L. Schlett, Fabian Bamberg, Esther Askani

**Affiliations:** 1 Department of Cardiology, Pneumology, Angiology, Acute Geriatrics and Intensive Care, Ortenau Klinikum, Klostenstraße 19, 77933 Lahr/Schwarzwald, Germany; 2 Department of Diagnostic and Interventional Radiology, Medical Center – University of Freiburg, Faculty of Medicine, University of Freiburg, Hugstetter Str. 55, 79106 Freiburg, Germany; 3 Department of Protestant Theology, Faculty of Theology, University of Heidelberg, Heidelberg, Germany; 4 University Emergency Center, Medical Center – University of Freiburg, Faculty of Medicine, University of Freiburg, Freiburg, Germany; 5 G.E.R.N. Research Center for Tissue Replacement, Regeneration and Neogenesis, Department of Orthopedics and Trauma Surgery, Medical Center – University of Freiburg, Faculty of Medicine, University of Freiburg, Freiburg, Germany; 6 Department of Anesthesiology and Intensive Care Medicine, Medical Center – University of Freiburg, Faculty of Medicine, University of Freiburg, Freiburg, Germany; 7 Department of Cardiovascular Surgery, Medical Center – University of Freiburg, Faculty of Medicine, University of Freiburg, Freiburg, Germany; 8 Department of General and Visceral Surgery, Medical Center – University of Freiburg, Faculty of Medicine, University of Freiburg, Freiburg, Germany; 9 Interdisciplinary Medical Intensive Care, Medical Center – University of Freiburg, Faculty of Medicine, University of Freiburg, Freiburg, Germany; 10 Clinic of Respiratory Medicine, Medical Center – University of Freiburg, Faculty of Medicine, University of Freiburg, Freiburg, Germany; 11 Division of Infectious Diseases, Department of Medicine II, Medical Center – University of Freiburg, Faculty of Medicine, University of Freiburg, Freiburg, Germany; 12 Institute of Virology, Medical Center – University of Freiburg, Faculty of Medicine, University of Freiburg, Freiburg, Germany

**Keywords:** ARDS, COVID-19, Vaccination, Hospitalization

## Abstract

This study aimed to analyze the effect of COVID-19 vaccination on the occurrence of ARDS in hospitalized COVID-19 patients. The study population of this retrospective, single-center cohort study consisted of hospitalized COVID-19 patients with known vaccination status and chest computed tomography imaging between July 2021 and February 2022. The impact of vaccination on ARDS in COVID-19 patients was assessed through logistic regression adjusting for demographic differences and confounding factors with statistical differences determined using confidence intervals and effect sizes. A total of 167 patients (69% male, average age 58 years, 95% CI [55; 60], 42% fully vaccinated) were included in the data analysis. Vaccinated COVID-19 patients had a reduced relative risk (RR) of developing ARDS (RR: 0.40, 95% CI [0.21; 0.62]). Consequently, non-vaccinated hospitalized patients had a 2.5-fold higher probability of developing ARDS. This risk reduction persisted after adjusting for several confounding variables (RR: 0.64, 95% CI [0.29; 0.94]) in multivariate analysis. The protective effect of COVID-19 vaccination increased with ARDS severity (RR: 0.61, 95% CI [0.37; 0.92]). Particularly, patients under 60 years old were at risk for ARDS onset and seemed to benefit from COVID-19 vaccination (RR: 0.51, 95% CI [0.20; 0.90]). COVID-19 vaccination showed to reduce the risk of ARDS occurrence in hospitalized COVID-19 patients, with a particularly strong effect in patients under 60 years old and those with more severe ARDS.

**Supplementary Information:**

The online version contains supplementary material available at 10.1007/s10238-023-01293-w.

## Background

COVID-19, caused by the SARS-CoV-2 coronavirus, was first reported in late 2019 and has since evolved into a global pandemic, with significant impacts on health, social, and economic well-being [1]. The development of vaccines has played a significant role in managing the pandemic [2].

Acute respiratory distress syndrome (ARDS) is a life-threatening condition characterized by widespread inflammation in the lungs and can be triggered by various events, including pneumonia, sepsis, or trauma [3, 4]. According to the Berlin definition, after exclusion of cardiac failure or fluid overload, ARDS is characterized by the onset of respiratory symptoms within 1 week of a known clinical insult or new/worsening respiratory symptoms and the presence of bilateral opacities on chest imaging. The definition also includes a minimum level of positive end-expiratory pressure (PEEP) and mutually exclusive PaO2/FiO2 thresholds for different levels of ARDS severity (mild, moderate, and severe) [5, 6].

The pathomechanisms of ARDS in COVID-19 are complex and still under investigation. However, it is known that the COVID-19 virus can trigger a dysregulated immune response, leading to a ‘cytokine storm.’ This excessive immune response can cause widespread inflammation and damage to the lung tissue leading to ARDS [7]. In addition, the COVID-19 virus can also directly infect endothelial cells, which line the blood vessels in the lungs causing endothelial dysfunction and increased vascular permeability further contributing to the development of ARDS [8, 9].

It is well-established that COVID-19 vaccination offers protection against COVID-19 infection, which could potentially lead to a reduction of ARDS among vaccinated individuals [10]. However, to date, no study has directly compared the prevalence of ARDS in hospitalized patients who have been vaccinated against those who have not.

Evaluating the impact of COVID-19 vaccination on ARDS is of paramount importance, especially in light of the ongoing debates surrounding vaccination. Comprehensive and robust evidence is required to conclusively address the potential benefits and risks associated with vaccination in the context of COVID-19.

In this study, we investigated the effect of COVID-19 vaccination on the occurrence of ARDS in patients hospitalized with a COVID-19 infection.

## Methods

### Study design and patient enrollment

This retrospective, single-center cohort study was conducted on a group of patients who were hospitalized due to COVID-19 infection at a high-level care hospital with the possibility of extracorporeal oxygenation. The vaccination status served as the intervention of interest, and the manifestation of ARDS served as the primary outcome.

**Fig. 1:**
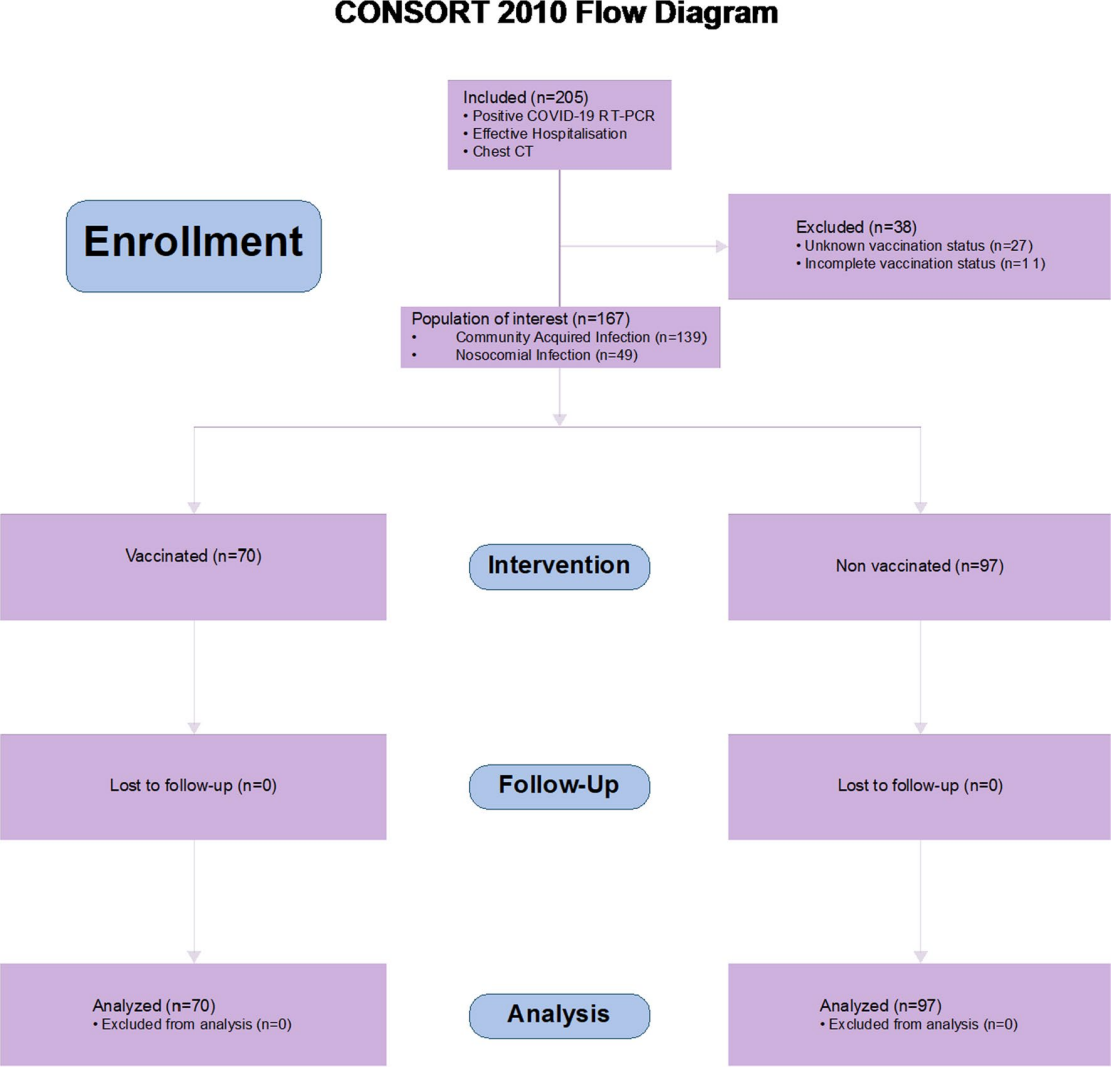
Flow diagram: enrollment of study participants

The hospital began documenting the vaccination history of in-patients from July 2021 onwards. The study included patients who were hospitalized with COVID-19 between July 1, 2021, and February 14, 2022. The inclusion criteria were a confirmed COVID-19 infection, evidenced by at least one positive RT-PCR test from a nasal or throat swab, and at least one chest CT scan during their hospital stay. Patients were excluded from the study, if there were incomplete data on their vaccination status, if they were partially vaccinated, or if they were under the age of 18 (Fig. 1).

### Vaccination status

The vaccination status was categorized into three groups: non-vaccinated, partially vaccinated, and fully vaccinated. Patients with explicit records on missing COVID-19 vaccination were classified as ‘non-vaccinated.’ Those who had received only one dose of vaccination or were diagnosed with COVID-19 less than 14 days after receiving their second dose were considered 'partially vaccinated.' Patients who tested positive for COVID-19 or showed symptoms at least 14 days after receiving their second vaccine dose were labeled as 'fully vaccinated.' Patients with no vaccination records or missing information about their last vaccination date were deemed to have an 'unknown vaccination status.'

### Data collection—demographic and clinical parameters

Upon identifying the study cohort in the electronic hospital information system, clinical data were collected from the electronic patient records. This data encompassed vaccination status, demographic information (age and sex), infection details (virus variant and onset of symptoms), symptoms (dyspnea, coughing, and fever), pre-existing conditions (body mass index (BMI), pregnancy, pre-existing disease, immunodeficiency, prediabetic metabolism, type 2 diabetes, hypertension, anemia, rheumatological disease, oncological disease, infectious disease, cardiac disease, vascular disease, pulmonary disease, neurological disease, liver disease, renal disease, thyroid disease, and organ transplant), laboratory parameters (C-reactive protein (CRP), procalcitonin (PCT), D-dimer, PTT, INR, pO2, and pCO2), treatment details (oxygen, non-invasive ventilation (NIV), high-flow oxygen therapy, intubation, extracorporeal membrane oxygenation (ECMO), extracorporeal lung support (ECLS), tracheotomy, intensive care unit (ICU) therapy, and ICU duration), and complications (sepsis, pulmonary superinfection, coagulopathy, renal failure, and mortality).

### ARDS and ARDS severity

According to the Berlin definition, after exclusion of cardiac failure or fluid overload, ARDS is characterized by the onset of respiratory symptoms within 1 week of a known clinical insult or new/worsening respiratory symptoms and the presence of bilateral opacities on chest imaging. ARDS severity is typically categorized in mild, moderate, and severe based on the PaO2/FiO2 ratio (the ratio of arterial oxygen partial pressure to fractional inspired oxygen) and the PEEP required: Mild ARDS has a PaO2/FiO2 ratio between 200 and 300 mmHg and a PEEP > 5-cm H2O; moderate ARDS has a PaO2/FiO2 ratio between 100 and 200 mmHg and a PEEP > 5-cm H2O; and severe ARDS has a PaO2/FiO2 ratio < 100 mmHg and a PEEP > 5-cm H2O [5, 6].

Information on the presence and severity of ARDS was collected from the patient history in the electronic medical record.

### CT examination

Chest CT examinations were performed with a high-resolution CT using Siemens Somatom Definition Flash (Siemens Healthineers, Erlangen, Germany). Tube current modulation CARE Dose4D at quality reference mAs of 100 mAs and automatic tube voltage setting with CARE kV at 120-kV reference with a collimation of 128 × 0.6 mm was used. Depending on the clinical question that needed to be answered, intravenous contrast agent was applied, as detailed elsewhere [11, 12]. In cases with follow-up CT scans, only the first acquired CT scan was evaluated.

The general extent of ARDS was semi-quantitatively scored for each pulmonary lobe: visual involvement of less than 1/3rd of lobar volume (score 1), visual involvement of 1/3rd to 2/3rd of lobar volume (score 2), and visual involvement of more than 2/3rd of lobar volume (score 3); the scores of the individual lobes were then added together (maximum possible score for both lungs being 15) [13, 14].

### Statistical analysis

Baseline differences in patients' characteristics between vaccinated and non-vaccinated patients were tested using logistic regression. Demographic parameters that were significantly different in both groups were used to adjust for confounding effects in the main analysis. Some demographic parameters, although rarely represented in the overall population, were still significantly different in both groups (patients with the omicron variant, healthy subjects, patients with immunodeficiency, oncological disease, vascular disease, neurological disease, organ transplant, and pregnancy). To avoid numerical instability in the regression analysis, a sub-analysis for all significant demographic parameters which showed fewer than 10 rare events was conducted. This sub-analysis was performed in the subgroup without these rare characteristics.

For the primary outcome, a logistic regression both with and without adjusting for confounding effects (age, type 2 diabetes, hypertension, cardiac disease, and pulmonary disease) was conducted. The same analysis in the subgroups excluding these rare events (virus variant, pre-existing disease, immunodeficiency, oncological disease, vascular disease, neurological disease, organ transplant, and pregnancy) was performed. Therefore, no statement about vaccine efficacy can be made for patients with these rare characteristics. In addition, a subgroup analysis for the primary outcome in a group of patients under 60 years old and over 60 years old was conducted. The efficacy of vaccination on ARDS was tested, correcting for confounding effects. Furthermore, a logistic ordinal regression on the effect of vaccination on increasing ARDS severity subgroups was performed. Logistic and linear regression analysis to assess the relationship between ARDS and clinical features, controlling for vaccination status was used. Additionally, where necessary, we controlled for the duration since symptom onset.

In order to identify relevant predictors for ARDS in COVID-19 patients, a logistic regression between each predictor and ARDS, adjusting for vaccination status, was conducted. To further optimize the model and select the relevant predictors for ARDS, a regularized logistic regression using least absolute shrinkage and selection operator (LASSO) regression was performed [15]. All effect sizes and adjusted effect sizes were computed with 95% confidence intervals using a bootstrapping method in addition to the generalized linear model.

For logistic regression, relative risk (RR) ratios and adjusted RR ratios where possible were calculated [16], and otherwise, odds ratios (OR) and adjusted ORs were computed. For linear regression, Cohen's d and adjusted Cohen's d were determined as relevant effect sizes. Significance was tested using confidence intervals (CI): RR ratios and ORs were deemed significant when the 95% CI did not include 1, and Cohen's d was deemed significant when the 95% CI did not include 0. Residual plots and q–q plots were used to check the independence of observations, unexplained trends in the residuals, linearity, normality, equality of variance, and outliers. Descriptive statistics were displayed: for continuous variables, the mean and 95% CIs were determined using bootstrapping; for categorical data, frequencies and proportions are displayed. Statistical analysis was performed using RStudio (v 2023.06.0 + 421, RStudio, Inc.).

## Results

### Demographic characteristics

The demographic characteristics of vaccinated and non-vaccinated patients are displayed in Table 1. Significant differences were observed between both groups for age (OR: 1.074, 95% CI [1.048; 1.102]), virus variant (OR 7.11, 95% CI [2.09; 33.00]), pre-existing diseases (OR: 7.75, 95% CI [2.87; 27.14]), immunodeficiency (OR: 4.78, 95% CI [1.96; 12.97]), type 2 diabetes (OR: 2.83, 95% CI [1.29; 6.43]), hypertension (OR: 4.07, 95% CI [2.12; 7.96]), oncological disease (OR: 6.50, 95% CI [2.59; 18.68]), cardiac disease (OR: 4.98, 95% CI [2.50; 10.26]), vascular disease (OR: 3.56, 95% CI [1.48; 9.27]), pulmonary disease (OR: 3.23, 95% CI [1.56; 6.85]), neurological disease (OR: 4.13, 95% CI [1.58; 12.18]), and organ transplant (OR: 10.83, 95% CI [2.85; 70.90]): Vaccinated patients were older and were suffering from pre-existing diseases more frequently. There were six cases of pregnancy in the non-vaccinated group and none in the vaccinated group.

**Table 1:** Demographic characteristics according to COVID-19 vaccination status

	All	Vaccination status (*n* = 167)		Odds ratio (+ / − 95% CI)
		Non-vaccinated (*n* = 97)	Vaccinated (*n* = 70)	
*General information*				
Age (years) (*n* = 167)	57.86	51.10	67.21	**1.074 (1.048; 1.102)**
	(55.33; 60.36)	(48.12; 54.04)	(63.97; 70.59)	
Sex (*n* = 167)				1.09 (0.56; 2.15)
Male	115 (69%)	66 (68%)	49 (70%)	
Female	52 (31%)	31 (32%)	21 (30%)	
Virus variant (*n* = 81)**				**7.11 (2.09; 33.00)**
Omicron	18 (22%)	3 (8%)	15 (37%)	
Delta	63 (78%)	37 (92%)	26 (63%)	
*Pre-existing conditions*				
BMI (*n* = 129)				0.75 (0.36; 1.57)
> 25 kg/m^2^	86 (67%)	50 (69%)	36 (42%)	
< 25 kg/m^2^	43 (33%)	22 (31%)	50 (58%)	
Pregnancy (*n* = 167)*				Infinite number
Yes	6 (3.5%)	6 (6%)	0 (0%)	
No	161 (96.5%)	91 (94%)	70 (100%)	
Pre-existing diseases (*n* = 167)**				**7.75 (2.87; 27.14)**
Yes	132 (79%)	66 (68%)	66 (94%)	
No	35 (21%)	31 (32%)	4 (6%)	
Immunodeficiency (through disease or medication) (*n* = 167)**				**4.78 (1.96; 12.97)**
Yes	26 (16%)	7 (7%)	19 (27%)	
No	141 (84%)	90 (93%)	51 (73%)	
Prediabetic metabolism (*n* = 167)*				0.45 (0.02; 3.63)
Yes	4 (2%)	3 (3%)	1 (1%)	
No	163 (98%)	94 (97%)	69 (99%)	
Type 2 diabetes (*n* = 167)				**2.83 (1.29; 6.43)**
Yes	32 (19%)	12 (12%)	20 (29%)	
No	135 (81%)	85 (88%)	50 (71%)	
Hypertension (*n* = 167)				**4.07 (2.12; 7.96)**
Yes	66 (40%)	25 (26%)	41 (59%)	
No	101 (60%)	72 (74%)	29 (41%)	
Anemia (*n* = 167)				1.32 (0.53; 3.21)
Yes	23 (14%)	12 (12%)	11 (16%)	
No	144 (86%)	85 (88%)	59 (84%)	
Rheumatological disease (*n* = 167)*				1.41 (0.37; 5.28)
Yes	10 (6%)	5 (5%)	5 (7%)	
No	157 (94%)	92 (95%)	65 (93%)	
Oncological disease (*n* = 167)**				**6.50 (2.59; 18.68)**
Yes	27 (16%)	6 (6%)	21 (30%)	
No	140 (84%)	91 (94%)	49 (70%)	
Infectious disease (*n* = 167)*				0.37 (0.05; 1.62)
Yes	9 (5%)	7 (7%)	2 (3%)	
No	158 (95%)	90 (93%)	68 (97%)	
Cardiac disease (*n* = 167)				**4.98 (2.50; 10.26)**
Yes	53 (32%)	17 (18%)	36 (51%)	
No	114 (68%)	80 (82%)	34 (49%)	
Vascular disease (*n* = 167)**				**3.56 (1.48; 9.27)**
Yes	25 (15%)	8 (8%)	17 (24%)	
No	142 (85%)	89 (92%)	53 (76%)	
Pulmonary disease (*n* = 167)				**3.23 (1.56; 6.85)**
Yes	41 (25%)	15 (15%)	26 (37%)	
No	126 (75%)	82 (85%)	44 (63%)	
Neurological disease (*n* = 167)**				**4.13 (1.58; 12.18)**
Yes	21 (13%)	6 (6%)	15 (21%)	
No	146 (87%)	91 (94%)	57 (79%)	
Liver disease (*n* = 167)*				0.57 (0.12; 2.15)
Yes	10 (6%)	7 (7%)	3 (5%)	
No	157 (94%)	90 (93%)	62 (95%)	
Renal disease (*n* = 167)				0.57 (0.12; 2.15)
Yes	38 (23%)	12 (12%)	26 (37%)	
No	129 (77%)	85 (88%)	44 (63%)	
Thyroid disease (*n* = 167)				1.11 (0.51; 2.38)
Yes	34 (20%)	19 (20%)	15 (21%)	
No	133 (80%)	78 (80%)	55 (79%)	
Organ transplant (*n* = 167)**				
Yes	15 (9%)	2 (2%)	13 (19%)	**10.83 (2.85; 70.90)**
No	152 (91%)	95 (98%)	57 (81%)	

Results in bold are statistically significant

*BMI* body mass index

*Rare characteristic but no significant difference between vaccination status and rare characteristic

**Rare characteristic and significant difference between vaccination status and rare characteristic

Virus variant, pre-existing disease, immunodeficiency, oncological disease, vascular disease, neurological disease, organ transplant, and pregnancy were rare characteristics in our dataset and therefore not suitable as confounding variables in a large multiple logistic regression model. Consequently, we used age, type 2 diabetes, hypertension, cardiac disease, and pulmonary disease as confounders in a comprehensive model and conducted a sub-analysis for virus variant, pre-existing disease, immunodeficiency, oncological disease, vascular disease, neurological disease, organ transplant, and pregnancy.

### Vaccination and ARDS

**Fig. 2:**
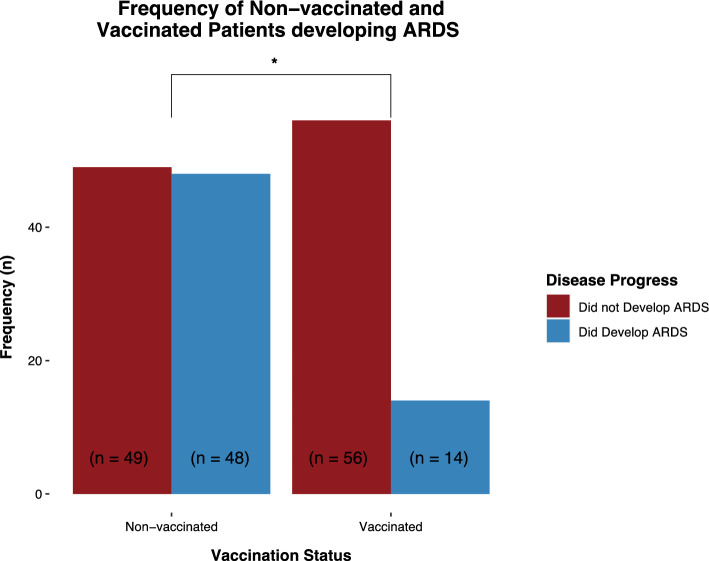
Frequency of ARDS in vaccinated and non-vaccinated hospitalized patients with COVID-19. Fully vaccinated patients and non-vaccinated patients were compared using logistic regression and relative risk ratio as effect size and bootstrapping methods to quantify uncertainty. About 95% CI was deemed significant. * Statistically significant difference

Without adjusting for confounding variables, vaccinated patients developed ARDS significantly less frequently than non-vaccinated patients (RR: 0.40, 95% CI [0.21; 0.62]). When adjusting for confounding variables such as age, type 2 diabetes, hypertension, cardiac disease, and pulmonary disease, the effect of vaccination remained significant (RR: 0.64, 95% CI [0.29; 0.94]) (Table 2 and Fig. 2).

**Table 2:** Vaccinated versus non-vaccinated hospitalized COVID-19 patients developing ARDS

	AII (*n* = 167)	Vaccination status		*P* vaIue	Estimate (+ / − 95% CI)	Risk ratio (+ / − 95% CI)
		Non-vaccinated	Vaccinated			
ARDS (*n* = 167)				Without correction for confounder variabies		
Yes	62 (37%)	48 (49%)	14 (20%)	**0.000157**	**− 1.36 (− 2.1; -0.67)**	**0.40 (0.21; 0.62)**
No	105 (63%)	49 (51%)	56 (80%)	With correction for confounder variabies		
				**0.017**	**− 1.02 (− 1.88; − 0.19)**	**0.64 (0.29; 0.94)**

Results in bold are statistically significant

The subgroup analyses excluded patients with rare events such as the omicron variant, healthy subjects, immunodeficiency, oncological disease, vascular disease, neurological disease, organ transplant, and pregnancy. The results remained significant in all subgroup analyses (Supplementary Table 1 and Supplementary Fig. 1).

### Vaccination and ARDS severity

**Fig. 3:**
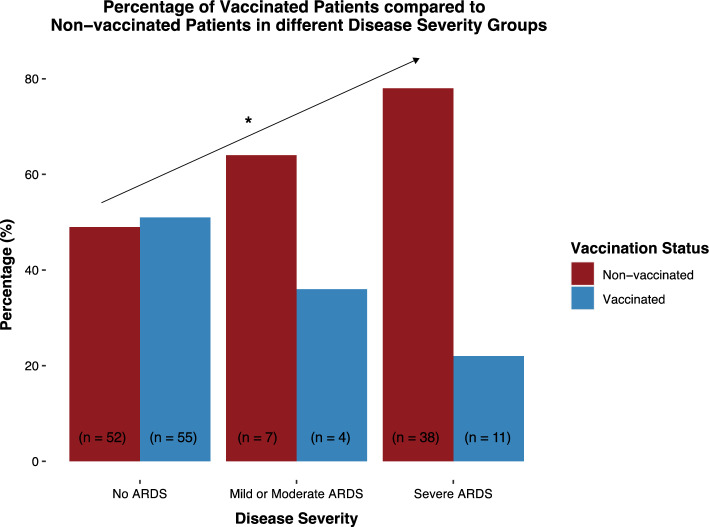
Percentage of vaccinated and non-vaccinated hospitalized COVID-19 patients in different ARDS disease severity groups. ARDS classified in mild, moderate, and severe according to the Berlin definition. Fully vaccinated patients and non-vaccinated patients were compared using ordered logistic regression and relative risk ratio as effect size and bootstrapping methods to quantify uncertainty. About 95% CI was deemed significant. * Statistically significant difference

Without adjusting for confounding variables, COVID-19 vaccination showed increasing protective effects with increasing severity of ARDS (RR: 0.63, 95% CI [0.41; 0.82]). After adjusting for confounding variables such as age, type 2 diabetes, hypertension, cardiac disease, and pulmonary disease, the effect remained significant (RR: 0.61, 95% CI [0.37; 0.92]) (Table 3 and Fig. 3).

**Table 3:** Vaccinated versus non-vaccinated hospitalized COVID-19 patients ordered in increasing ARDS severity groups

	Disease severity			Risk ratio (+ / − 95% Cl)
	No ARDS	Mild and moderate ARDS	Severe ARDS	
Vaccination status (*n* = 167)				Without correction for confounder variables
Vaccinated	55 (51%)	4 (36%)	11 (22%)	**0.63 (0.41; 0.82)**
Non-vaccinated	52 (49%)	7 (64%)	38 (78%)	With correction for confounder variables
				**0.61 (0.37; 0.92)**

ARDS classified in mild, moderate, and severe according to the Berlin definition. Results in bold are statistically significant

**Fig. 4:**
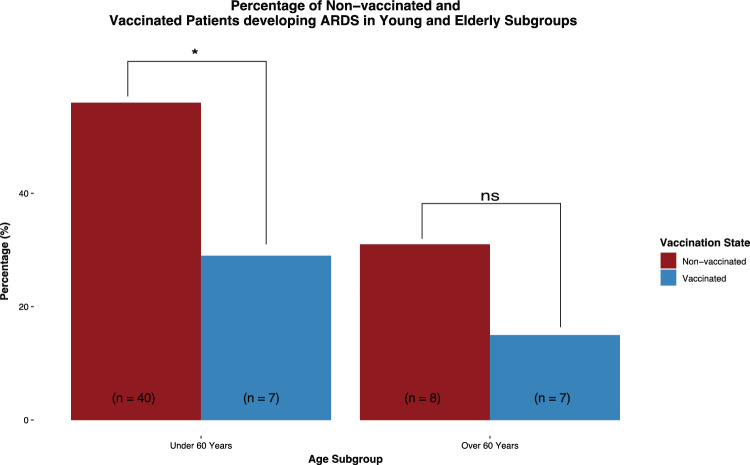
Percentage of vaccinated and non-vaccinated hospitalized COVID-19 patients developing ARDS in young and elderly subgroups. Fully vaccinated patients and non-vaccinated patients were compared using logistic regression and relative risk ratio as effect size and bootstrapping methods to quantify uncertainty. About 95% CI was deemed significant. * Statistically significant difference; ns statistically non-significant

Patients under 60 years old developed ARDS significantly more often than patients over 60 years old (OR: 0.956, 95% CI [0.934; 0.976]). This difference persisted when controlling for vaccination status (OR: 0.966, 95% CI [0.943; 0.089]) (Table 4). Therefore, we evaluated the effect of vaccination in patients under 60 years old and patients over 60 years old separately. Patients under 60 years old developed ARDS less frequently when they were vaccinated (RR: 0.51, 95% CI [0.20; 0.90]), whereas patients over 60 years old did not show a significant effect of the vaccination on the onset of ARDS (Table 5 and Fig. 4).

**Table 4:** Predictive risk factors for hospitalized COVID-19 patients developing ARDS

		ARDS status		Without correction for vaccination status	With correction for vaccination status
		No ARDS	ARDS	Odds ratio (+ / − 95% CI)	Adj odds ratio (+ / − 95% CI)
Age (years *n* = 167)	57.85 (55.34; 60.29)	62.09 (58.89; 65.12)	50.67 (47.36; 54.21)	**0.956 (0.934; 0.976)**	**0.966 (0.943; 0.989)**
Sex *n* = (167)				0.81 (0.41; 1.61)	0.82 (0.40; 1.68)
Male	115 (69%)	74 (70%)	41 (66%)		
Female	52 (31%)	31 (30%)	21 (34%)		
Virus variant (*n* = 81)				**0.12 (0.00; 0.68)**	**0.16 (0.00; 0.95)**
Omicron	18 (22%)	17 (28%)	1 (5%)		
Delta	63 (78%)	43 (72%)	20 (95%)		
BMI (*n* = 129)				**2.35 (1.03; 5.78)**	2.29 (0.97; 5.81)
> 25 kg/m^2^	86 (67%)	53 (61%)	33 (79%)		
< 25 kg/m^2^	43 (33%)	34 (39%)	9 (21%)		
Pregnancy (*n* = 167)				Infinite number	Infinite number
Yes	6 (3.5%)	0 (0%)	6 (10%)		
No	161 (96.5%)	105 (100%)	56 (90%)		
Pre-existing diseases (*n* = 167)				0.472 (0.22; 1.00)	0.73 (0.0.32; 1.63)
Yes	132 (79%)	88 (84%)	44 (71%)		
No	35 (21%)	17 (16%)	18 (29%)		
Immunodeficiency (through disease or medication) (*n* = 167)				**0.35 (0.11; 0.91)**	0.53 (0.16; 1.49)
Yes	26 (16%)	21 (20%)	5 (8%)		
No	141 (84%)	84 (80%)	57 (92%)		
Prediabetic metabolism (*n* = 167)				0.55 (0.02;4.46)	0.42 (0.02; 3.68)
Yes	4 (2%)	3 (3%)	1 (2%)		
No	163 (98%)	102 (97%)	61 (98%)		
Type 2 diabetes (*n* = 167)				1.02 (0.44; 2.23)	1.51 (0.62; 3.63)
Yes	32 (19%)	20 (19%)	12 (19%)		
No	135 (81%)	85 (81%)	50 (81%)		
Hypertension (*n* = 167)				**0.48 (0.24; 0.93)**	0.70 (0.33; 0.1.45)
Yes	66 (40%)	48 (46%)	18 (29%)		
No	101 (60%)	57 (54%)	44 (71%)		
Anemia (*n* = 167)				0.70 (0.25; 1.77)	0.75 (0.26; 1.98)
Yes	23 (14%)	16 (15%)	7 (11%)		
No	144 (86%)	89 (85%)	55 (89%)		
Rheumatological disease (*n* = 167)				0.40 (0.05; 1.67)	0.42 (0.05; 1.87)
Yes	10 (6%)	8 (8%)	2 (3%)		
No	157 (94%)	97 (92%)	60 (97%)		
Oncological disease (*n* = 167)				**0.17 (0.03; 0.52)**	**0.26 (0.05; 0.84)**
Yes	27 (16%)	24 (23%)	3 (5%)		
No	140 (84%)	81 (77%)	59 (95%)		
Infectious disease (n = 167)				0.83 (0.17; 3.30)	0.62 (0.12; 2.61)
Yes	9 (5%)	6 (6%)	3 (5%)		
No	158 (95%)	99 (94%)	59 (95%)		
Cardiac disease (*n* = 167)				**0.32 (0.14; 0.67)**	0.47 (0.20; 1.04)
Yes	53 (32%)	42 (40%)	11 (18%)		
No	114 (68%)	63 (60%)	51 (82%)		
Vascular disease (*n* = 167)				**0.11 (0.01; 0.42)**	**0.15 (0.02; 0.57)**
Yes	25 (15%)	23 (21%)	2 (3%)		
No	142 (85%)	82 (78%)	60 (97%)		
Pulmonary disease (*n* = 167)				0.96 (0.45; 1.99)	1.50 (0.66; 3.42)
Yes	41 (25%)	26 (25%)	15 (24%)		
No	126 (75%)	79 (75%)	47 (76%)		
Neurological disease (*n* = 167)				0.48 (0.15; 1.32)	0.73 (0.22; 2.16)
Yes	21 (13%)	16 (15%)	5 (8%)		
No	146 (87%)	89 (85%)	57 (92%)		
Liver disease (*n* = 167)				0.71 (0.14; 2.66)	0.58 (0.11; 2.31)
Yes	10 (6%)	7 (7%)	3 (5%)		
No	157 (94%)	98 (93%)	59 (95%)		
Renal disease (*n* = 167)				**0.37 (0.14; 0.83)**	0.53 (0.20; 1.28)
Yes	38 (23%)	30 (29%)	8 (13%)		
No	129 (77%)	75 (71%)	54 (87%)		
Thyroid disease (*n* = 167)				1.06 (0.47; 2.28)	1.10 (0.48; 2.49)
Yes	34 (20%)	21 (20%)	13 (21%)		
No	133 (80%)	84 (80%)	49 (79%)		
Organ transplant (*n* = 167)				0.39 (0.08; 1.30)	0.75 (0.15; 2.78)
Yes	15 (9%)	12 (11%)	3 (5%)		
No	152 (91%)	93 (89%)	59 (95%)		

Results in bold are statistically significant

*BMI* body mass index

**Table 5:** Vaccinated versus non-vaccinated hospitalized COVID-19 patients developing ARDS in young (< 60 years) and elderly subgroups (> 60 years)

	AII (*n* = 167)	Vaccination status		Risk ratio (+ / − 95% CI)
		Non-vaccinated	Vaccinated	
ARDS in patients < 60 years (*n* = 95)				**0.51 (0.20; 0.90)**
Yes	47 (49%)	40 (56%)	7 (29%)	
No	48 (51%)	31 (44%)	17 (71%)	
ARDS in patients > 60 years (*n* = 72)				0.49 (0.16; 1.33)
Yes	15 (21%)	8 (31%)	7 (15%)	
No	57 (79%)	18 (69%)	39 (85%)	

Results in bold are statistically significant

### ARDS and predictive risk factors

In order to evaluate the risk factors for developing ARDS in COVID-19 patients, we performed a logistic regression between ARDS and the patients' characteristic variables, controlling for vaccination status (Table 4). After adjusting for vaccination status, the following characteristics remained significant for developing ARDS: Older patients had lower odds (OR: 0.966, 95% CI [0.943; 0.989]), patients with the omicron variant had lower odds (OR: 0.16, 95% CI [0.00; 0.95]), patients with oncological disease had lower odds (OR: 0.26, 95% CI [0.05; 0.84]), and patients with vascular disease had lower odds (OR: 0.15, 95% CI [0.02; 0.57]). All six pregnant women developed ARDS, making it impossible to compute ORs with CIs; however, this suggests a strong relationship. Therefore, younger patients, patients with the delta variant, patients without oncological disease, patients without vascular disease, and pregnant patients were more prone to developing ARDS. Conversely, older patients, patients with the omicron variant, patients with oncological disease, patients with vascular disease, and non-pregnant patients were less prone to developing ARDS.

Other characteristics had a significant relationship with ARDS in univariate analysis, but the relationship disappeared after adjusting for vaccination status: Obese patients had higher odds (OR: 2.35, 95% CI [1.03; 5.78]), patients with immunodeficiency had lower odds (OR: 0.35, 95% CI [0.11; 0.91]), patients with hypertension had lower odds (OR: 0.48, 95% CI [0.24; 0.93]), patients with cardiac disease had lower odds (OR: 0.32, 95% CI [0.14; 0.67]), and patients with renal disease had lower odds (OR: 0.37, 95% CI [0.14; 0.83]). There was no association between ARDS and type 2 diabetes mellitus (OR: 1.02, 95% CI [0.44; 2.23]).

This standard approach of assessing each variable separately has some limitations because these variables have unknown amounts of correlation among each other. Therefore, we performed a regularized logistic regression using LASSO regression incorporating all variables, the model confirmed our prior results that younger patients, patients without immunodeficiency, patients without oncological disease, patients without cardiac disease, patients without vascular disease, patients who were non-vaccinated, and pregnant women were associated with a higher frequency of ARDS.

Furthermore, we investigated clinical features associated with COVID-19 ARDS in our dataset and adjusted the results for vaccination (Table 6): COVID-19 ARDS was associated with increased dyspnea (OR: 16.8, 95% CI [3.3; 307]), amount of oxygen therapy (OR: 1.20, 95% CI [1.08; 1.34]), NIV (OR: 110, 95% CI [34; 473]), high-flow oxygen therapy (OR: 36, 95% CI [13; 118]), intubation and invasive ventilation (OR: 71, 95% CI [25; 246]), ECMO-ECLS (all 25 ECMO-ECLS patients were in the ARDS group, therefore, computing OR and CI was not possible), tracheotomy (OR: 18, 95% CI [5; 84]), ICU therapy (OR: 139, 95% CI [37; 918]), ICU duration (OR: 1.13, 95% CI [1.08; 1.20]), higher pO2 (OR: 1.025, 95% CI [1.012; 1.041]), higher pCO2 (OR: 1.06, 95% CI [1.03; 1.10]), higher mortality (OR: 12.81, 95% CI [5.18; 35.80]), more sepsis (OR: 33, 95% CI [9.1; 219]), higher CRP level (OR: 1.005, 95% CI [1.001; 1.01]), more pulmonary superinfections (OR: 12.67, 95% CI [5.64; 31.06]), higher D-dimer levels (OR: 1.09, 95% CI [1.03; 1.17]), more coagulopathy (OR: 18.7, 95% CI [3.36; 353]), and more renal failure (OR: 2.76, 95% CI [1.38; 5.61]). ARDS was associated with higher PCT (OR: 1.31, 95% CI [1.05; 1.73]), but this effect was not significant when adjusting for vaccination status.

**Table 6:** Clinical characteristics of hospitalized COVID-19 patients developing ARDS

		ARDS status		Without correction for vaccination status	With correction for vaccination status
		No ARDS	ARDS	Odds ratio (+ / − 95% CI)	Adj odds ratio (+ / − 95% CI)
Dyspnea (*n* = 165)				**19.9 (4.0; 362.0)**	**16.8 (3.3; 307)**
Yes	138 (84%)	78 (75%)	60 (98%)		
No	27 (16%)	26 (25%)	1 (2%)		
Coughing (*n* = 150)				1.28 (0.64; 2.62)	1.43 (0.69; 3.02)
Yes	90 (60%)	58 (58%)	32 (64%)		
No	60 (40%)	42 (42%)	18 (36%)		
Fever (*n* = 149)				1.72 (0.83; 3.69)	2.04 (0.95; 4.56)
Yes	93 (62%)	59 (58%)	34 (64%)		
No	56 (38%)	42 (42%)	14 (36%)		
Oxygen (l/min *n* = 110)	4.20 (3.42; 5.03)	3.36 (2.58; 4.19)	7.34 (5.26; 9.30)	**1.19 (1.08; 1.33)**	**1.20 (1.08; 1.34)**
NIV (*n* = 153)				**84 (29;292)**	**110 (34; 473)**
Yes	56(37%)	10 (10%)	46 (90%)		
No	97 (63%)	92 (90%)	5 (10%)		
High flow (*n* = 147)				**28 (11; 79)**	**36 (13; 118)**
Yes	43 (29%)	9 (9%)	34 (74%)		
No	104 (71%)	92 (91%)	12 (26%)		
Intubation (*n* = 165)				**80 (29; 271)**	**71 (25; 246)**
Yes	54 (33%)	5 (5%)	49 (80%)		
No	111 (67%)	99 (95%)	12 (20%)		
ECMO-ECLS (*n* = 165)				Infinite number	Infinite number
Yes	25 (15%)	0 (0%)	25 (40%)		
No	140 (85%)	103 (100%)	37 (60%)		
Tracheotomy (*n* = 163)				**20 (6; 88)**	**18 (5; 84)**
Yes	25 (15%)	3 (3%)	22 (37%)		
No	138 (85%)	101 (97%)	37 (63%)		
Quantitative score of chest CT lesions (*n* = 167)	7.31 (6.61; 7.99)	4.49 (4.20; 5.54)	11.38 (10.57; 12.24)	**1.63 (1.43; 1.92)**	**1.62 (1.41; 1.92)**
ICU therapy (*n* = 167)				**135 (37; 814)**	**139 (37; 918)**
Yes	79 (47%)	19 (18%)	60 (97%)		
No	88 (53%)	86 (82%)	2 (3%)		
ICU duration (days *n* = 158)	8.30 (6.01; 10.95)	2.14 (0.67; 4.15)	19.83 (15.12; 24.51)	**1.14 (1.08; 1.21)**	**1.13 (1.08; 1.20)**
pO2 (mmHg *n* = 151)	54.40 (50.09; 59.14)	47.54 (41.64; 53.79)	64.53 (60.14; 69.27)	**1.024 (1.011; 1.039)**	**1.025 (1.012; 1.041)**
pCO2 (mmHg *n* = 156)	42.96 (40.89; 45.13)	39.13 (37.61; 40.42)	48.92 (44.41; 53.57)	**1.06 (1.03; 1.10)**	**1.06 (1.03; 1.10)**
Mortality (*n* = 165)				**10.87 (4.70; 27.77)**	**12.81 (5.18; 35.80)**
Yes	37 (22%)	8 (8%)	29 (48%)		
No	128 (78%)	96 (92%)	32 (52%)		
Sepsis (*n* = 165)				**36.4 (10.2; 233)**	**33 (9.1; 219)**
Yes	28 (17%)	2 (2%)	26 (42%)		
No	137 (83%)	101 (98%)	36 (58%)		
C-reactive protein (mg/l *n* = 144)	102 (87; 117)	99 (75; 107)	131 (101; 162)	**1.004 (1.001; 1.008)**	**1.005 (1.001; 1.01)**
Procalcitonin (mug/l *n* = 140)	0.78 (0.53; 1.09)	0.52 (0.31; 0.81)	1.23 (0.69; 1.85)	**1.31 (1.05; 1.73)**	1.25 (0.99; 1.67)
Pulmonary superinfection (*n* = 159)				**10.75 (5.06; 24.52)**	**12.67 (5.64; 31.06)**
Yes	78 (49%)	29 (29%)	49 (82%)		
No	81 (51%)	70 (71%)	11 (18%)		
D-dimer (mg/l *n* = 138)	4.82 (3.54; 6.26)	2.85 (2.00; 3.98)	8.40 (5.41; 11.60)	**1.10 (1.04; 1.18)**	**1.09 (1.03; 1.17)**
PTT (sec *n* = 160)	36.61 (34.50; 39.18)	36.47 (33.60; 40.25)	36.83 (34.16; 40.29)	1.00 (0.97; 1.02)	1.00 (0.97; 1.02)
INR (*n* = 160)	1.16 (1.09; 1.26)	1.16 (1.09; 1.25)	1.17 (1.05; 1.39)	1.03 (0.52; 1.88)	1.08 (0.57; 2.15)
Coagulopathy (*n* = 165)				**21.9 (4.1; 407)**	**18.7 (3.36; 353)**
Yes	12 (7%)	1 (1%)	11 (18%)		
No	153 (93%)	102 (99%)	51 (82%)		
Renal failure (*n* = 166)				**2.58 (1.34; 5.02)**	**2.76 (1.38; 5.61)**
Yes	60 (36%)	29 (28%)	31 (50%)		
No	106 (64%)	75 (72%)	31 (50%)		

Results in bold are statistically significant

*NIV* non-invasive ventilation, *ECMO-ECLS* extracorporeal membrane oxygenation and extracorporeal life support, *ICU* intensive care unit, *pO2* partial pressure of oxygen, *pCO2* partial pressure of CO_2_, *PTT* partial thromboplastin time, and *INR* international normalized ratio

## Discussion

In this retrospective single-cohort study, vaccinated hospitalized COVID-19 patients were less likely to develop ARDS. This effect persisted even when controlling for a variety of confounding variables such as age, virus variant, and pre-existing diseases. The protective effect of vaccination was particularly strong in younger patients and increased with disease severity. Vaccinated patients had less severe outcomes of their COVID-19 infection. Older age and diseases associated with an impaired immune system reduced the odds of developing ARDS in COVID-19. The clinical characteristics of COVID-19 were typical of a severe disease course.

Our finding that hospitalized patients with COVID-19 who were vaccinated developed ARDS less frequently than those who were not vaccinated aligns with existing knowledge that COVID-19 vaccination reduces mortality and hospitalization in the overall population [10, 17]. However, our study is the first to report an association between COVID-19 vaccination and a reduced ARDS occurrence in hospitalized patients.

Our study specifically examined hospitalized COVID-19 patients, even those who had received vaccination. Unlike many studies that looked at how COVID-19 vaccination affects the overall population, we assessed only those already hospitalized. Therefore, we focused on how vaccination may protect hospitalized patients. This is crucial because it enables prognostic insights into how vaccination might influence the risk of these patients developing ARDS.

Younger patients developed ARDS more frequently than older patients in our study. This finding may seem surprising, as more severe outcomes and higher mortality rates are typically associated with older age [18]. But, it is important to note that both groups of older and younger patients in our study were relatively older compared to the non-hospitalized population. Very young individuals with COVID-19 may possess a robust and well-adapted immune system, which significantly reduces the likelihood of hospitalization [19]. ARDS is thought to be a result of an overwhelming and dysregulated response of the immune system [20]. Middle-aged individuals (the younger group in our study) may have an immune system that struggles to adapt to COVID-19, leading to an overwhelming immune response, a cytokine storm, and ARDS. It is possible that the diminished capacity for cytokine production in older patients [21, 22] may prevent the cytokine storm that is characteristic of ARDS.

Consistent with these results, patients with significant immune system impairment (those with immunodeficiency or oncological diseases) had reduced odds of developing ARDS in COVID-19. Similar results have been found in previous studies [23]. However, pregnancy appears to be a predisposing risk factor for ARDS in COVID-19, likely due to an enhanced immune response to viral infections [24]. Surprisingly, vascular disease and cardiac disease emerged as protective factors against the development of ARDS in COVID-19. These diseases might be associated with a weaker immune response, but evidence is currently lacking.

The pathomechanism of COVID-19 infection seems to involve two phases. The first phase is associated with strong virus-induced immunosuppression, and the second phase involves a reactive, dysregulated overreaction of the immune system, particularly found in severe cases of COVID-19 [25–27]. To develop this second phase of immune overreaction, a sufficiently functioning immune system is required. The virus-induced immunosuppression in the first phase of COVID-19 infection is dependent on the viral load [26]. Vaccination decreases the viral load in the first phase of the infection, likely reducing the virus-induced immunosuppression [28, 29], and thereby reducing the risk of a secondary overreaction of the immune system.

Assuming that vaccination reduces the risk of a secondary overreaction, particularly the younger patients in our study who had higher odds of developing ARDS may benefit from the vaccination. In our study, patients under the age of 60 years were more likely to benefit from vaccination concerning the development of ARDS than patients over 60 years. Therefore, our results are coherent with the postulated theory. If patients under 60 years old benefit more from vaccination than very old patients, this would have implications for the vaccination strategy in a future pandemic. However, even if patients under 60 years old are more prone to ARDS and benefit more from the vaccination effect on ARDS onset, it does not necessarily mean that younger patients die more often than older patients. In fact, it seems that older patients die more often from COVID-19 [30], probably because of direct effects of the viral infection and not because of an overreaction of the immune system.

In order to discover underlying molecular mechanisms that could lead to an ARDS risk reduction, bronchial epithelial cell gene expression studies can provide valuable insights; molecular and immunological insights could enable risk stratification and personalized interventions for individuals at higher risk for the development of ARDS. Explainable artificial intelligence plays a crucial role in deciphering complex gene expression patterns and guiding clinical decisions in several ways and integrating multi-omics data in understanding COVID-19 pathogenesis can contribute to advancing our knowledge of the disease. Karami et al. used the bioinformatics tool weighted gene co-expression network analysis (WGCNA) for the identification of gene modules and networks within biological systems and were able to discover novel candidate drugs, which could potentially be used to treat COVID-19 patients [31]. By combining information on vaccination status and gene expression personalized treatment approaches may be achieved.

ARDS caused by COVID-19 infection exhibits a specific pattern of clinical features that differ from ARDS caused by other diseases [32]. Overall, our findings align with those of the previous studies: ARDS patients exhibited more clinical symptoms such as dyspnea, they required more frequently and longer ICU therapies, and they experienced higher mortality rates. Additionally, superinfections were more common in COVID-19 ARDS patients than in non-ARDS patients. It has to be reflected that these complications may be associated with long-term complications associated with COVID-19.

COVID-19 is associated with a more localized type of disseminated intravascular coagulation, typically found in sepsis and ARDS, known as pulmonary intravascular coagulation. This condition is typically associated with higher D-dimer levels and normal PTT and INR values [26]. This is believed to be mediated through direct viral damage to the vascular endothelium in the infected organ [33]. Consistent with this hypothesis, we observed higher D-dimer levels and more instances of coagulopathy in COVID-19 ARDS patients compared to non-ARDS patients.

Of course, there are several important areas for future research on the subject, including the durability of protective vaccination effects, immune memory dynamics, and personalized treatment algorithms.

## Limitations

We conducted a retrospective single-center cohort study. Due to the retrospective nature of the study, the risk for confounding factors and incomplete or biased information is higher than in a prospective cohort study. However, we meticulously controlled and adjusted our results for all potential confounding variables. Furthermore, in order to overcome the curse of high dimensionality, we used regularized logistic regression to control for relevant confounder. Potential variability in CT scanning practices may have occurred. However, we do not expect implications on ARDS severity assessment, since different uses of contrast agent should not have an impact on the assessment of lung parenchymal involvement. Of course, it has to be considered, that being conducted at a single high-level care hospital, the generalizability of study findings to a broader population is limited, as patient demographics and management strategies may vary across different health-care settings and specific confounders such as socioeconomic status could impact the observed associations. However, the study was conducted in a country, in which health-care access for everybody is not an issue due to a system of statutory health insurance. Certain confounding variables arose due to political decisions in the health-care system. In Germany, older people were prioritized for vaccination, hence the importance of identifying and adjusting for these confounding variables. Although a cohort of nearly 170 patients was sufficient to study most effects in our population, we could not fully analyze rare characteristics or events. For instance, our sample included only six pregnant women, so it is not possible to make definitive statements about the effect of vaccination in some subpopulations or subgroups. Furthermore, due to the small sample size, we had to exclude partially vaccinated individuals, which may introduce selection bias. Nevertheless, the effect of vaccination on the overall population remained significant despite correcting for all confounding variables. Also, ARDS severity categorization based on the PaO2/FiO2 ratio and PEEP may not take into account additional factors influencing ARDS severity, such as comorbidities and individual patient responses. However, predictive risk factors for COVID-19 patients developing ARDS and clinical responses were additionally analyzed in detail. Moreover, the virus is a dynamically mutating organism, which complicates the generalization of our results to new mutations and other strains in the future years. However, an iterative analysis of many independent studies at different given time points may enable the extrapolation of deeper principles in the future meta-analysis studies. Moreover, multicenter observations on the subject should be considered to enhance the external validity of findings and accommodate diverse patient populations and management strategies. In our study, an ARDS reduction was observed in vaccinated hospitalized patients. However, there is variation in the relationship of new variants and ARDS on the one hand, and on the other hand, vaccination effectiveness may vary in new variants. Because of the very dynamic characteristics of this pandemic, iterative research is needed.

## Conclusions

COVID-19 vaccination showed to reduce the risk of ARDS occurrence in hospitalized COVID-19 patients, with a particularly strong effect in patients under 60 years old and those with more severe ARDS.

ARDS was more prevalent in younger COVID-19 patients (around 50 years of age) than in older COVID-19 patients (around 63 years of age). Consequently, especially COVID-19 patients under 60 years old may benefit from the protective vaccination effects.

## Supplementary Information

Below is the link to the electronic supplementary material.

Primary outcome. Percentage of non-vaccinated and vaccinated hospitalized COVID-19 patients developing ARDS in subgroups adjusted for different confounding factors
Supplementary file1 (EPS 941 kb)

Primary outcome. Vaccinated versus non-vaccinated hospitalized COVID-19 patients developing ARDS in subgroups adjusted for different confounding factors. Results highlighted in blue are statistically significant
Supplementary file2 (DOCX 17 kb)

## Data Availability

The datasets used and analyzed during the current study are available from the corresponding author on reasonable request.

## Abbreviations

ARDS: Acute respiratory distress syndrome
BMI: Body mass index
CI: Confidence interval
CRP: C-reactive protein
CT: Computed tomography
COVID-19: Coronavirus disease-19
ECLS: Extracorporeal lung support
ECMO: Extracorporeal membrane oxygenation
FiO2: Fraction of inspired oxygen
ICU: Intensive care unit
INR: International normalized ratio
LASSO: Least absolute shrinkage and selection operator
NIV: Non-invasive ventilation
OR: Odds ratio
PaO2: Partial pressure of oxygen
PCT: Procalcitonin
PEEP: Positive end-expiratory pressure
PTT: Partial thromboplastin time
RR: Relative risk

## References

[1] Karim SSA, Karim QA. Omicron SARS-CoV-2 variant: a new chapter in the COVID-19 pandemic. Lancet. 2021;398:2126–8. 34871545 10.1016/S0140-6736(21)02758-6 PMC8640673

[2] Fontanet A, Autran B, Lina B, Kieny MP, Karim SSA, Sridhar D. SARS-CoV-2 variants and ending the COVID-19 pandemic. Lancet. 2021;397:952–4. 33581803 10.1016/S0140-6736(21)00370-6 PMC7906631

[3] Standiford TJ, Ward PA. Therapeutic targeting of acute lung injury and acute respiratory distress syndrome. Transl Res. 2016;167:183–91. 26003524 10.1016/j.trsl.2015.04.015 PMC4635065

[4] Wu C, Chen X, Cai Y, Xia J, Zhou X, Xu S, et al. Risk factors associated with acute respiratory distress syndrome and death in patients with coronavirus disease 2019 pneumonia in Wuhan China. JAMA Intern Med. 2020;180(934):43. 32167524 10.1001/jamainternmed.2020.0994 PMC7070509

[5] Ferguson ND, Fan E, Camporota L, Antonelli M, Anzueto A, Beale R, et al. The Berlin definition of ARDS: an expanded rationale, justification, and supplementary material. Intensive Care Med. 2012;38:1573–82. 10.1007/s00134-012-2682-1. 22926653 10.1007/s00134-012-2682-1

[6] Ranieri VM, Rubenfeld GD, Thompson BT, Ferguson ND, Caldwell E, Fan E, et al. Acute respiratory distress syndrome: the Berlin definition. JAMA. 2012;307:2526–33. 22797452 10.1001/jama.2012.5669

[7] Matthay MA, Zemans RL, Zimmerman GA, Arabi YM, Beitler JR, Mercat A, et al. Acute respiratory distress syndrome. Nat Rev Dis Prim. 2019;5:1–22. 30872586 10.1038/s41572-019-0069-0 PMC6709677

[8] Merad M, Martin JC. Author correction: pathological inflammation in patients with COVID-19: a key role for monocytes and macrophages. Nat Rev Immunol. 2020;20:448. 10.1038/s41577-020-0331-4. 32488203 10.1038/s41577-020-0353-y PMC7265161

[9] Li CX, Noreen S, Zhang LX, Saeed M, Wu PF, Ijaz M, et al. A critical analysis of SARS-CoV-2 (COVID-19) complexities, emerging variants, and therapeutic interventions and vaccination strategies. Biomed Pharmacother. 2022;146:112550. 34959116 10.1016/j.biopha.2021.112550 PMC8673752

[10] Polack FP, Thomas SJ, Kitchin N, Absalon J, Gurtman A, Lockhart S, et al. Safety and efficacy of the BNT162b2 mRNA Covid-19 vaccine. N Engl J Med. 2020;383:2603–15. 10.1056/nejmoa2034577. 33301246 10.1056/NEJMoa2034577 PMC7745181

[11] Askani E, Mueller-Peltzer K, Madrid J, Knoke M, Hasic D, Schlett CL, et al. Pulmonary computed tomographic manifestations of COVID-19 in vaccinated and non-vaccinated patients. Sci Rep. 2023;131:1–13. 10.1038/s41598-023-33942-1 PMC10134716 37105996

[12] Askani E, Mueller-Peltzer K, Madrid J, Knoke M, Hasic D, Bamberg F, et al. Computed tomographic imaging features of COVID-19 pneumonia caused by the delta (B.1.617.2) and omicron (B.1.1.529) variant in a German nested cohort pilot study group. Tomography. 2022;8:2435–49. 36287801 10.3390/tomography8050202 PMC9607412

[13] von Spee-Mayer C, Echternach C, Agarwal P, Gutenberger S, Soetedjo V, Goldacker S, et al. Abatacept use is associated with steroid dose reduction and improvement in fatigue and CD4-dysregulation in CVID patients with interstitial lung disease. J Allergy Clin Immunol Pract. 2021;9:760-770.e10. 33223097 10.1016/j.jaip.2020.10.028

[14] De Jong PA, Vos R, Verleden GM, Vanaudenaerde BM, Verschakelen JA. Thin-section computed tomography findings before and after azithromycin treatment of neutrophilic reversible lung allograft dysfunction. Eur Radiol. 2011;21:2466–74. 10.1007/s00330-011-2224-1. 21792614 10.1007/s00330-011-2224-1 PMC3217146

[15] Friedman J, Hastie T, Tibshirani R. Regularization paths for generalized linear models via coordinate descent. J Stat Softw. 2010;33:1. 20808728 PMC2929880

[16] Nakagawa S, Cuthill IC. Effect size, confidence interval and statistical significance: a practical guide for biologists. Biol Rev. 2007;82:591–605. 10.1111/j.1469-185X.2007.00027.x. 17944619 10.1111/j.1469-185X.2007.00027.x

[17] Bahl A, Johnson S, Maine G, Garcia MH, Nimmagadda S, Qu L, et al. Vaccination reduces need for emergency care in breakthrough COVID-19 infections: a multicenter cohort study. Lancet Reg Heal-Am. 2021;4:100065. 10.1016/j.lana.2021.100065 PMC8428472 34522911

[18] Sundaram SS, Melquist S, Kalgotra P, Srinivasan S, Parasa S, Desai M, et al. Impact of age, sex, race, and regionality on major clinical outcomes of COVID-19 in hospitalized patients in the United States. BMC Infect Dis. 2022;22:1–10. 10.1186/s12879-022-07611-z. 35906558 10.1186/s12879-022-07611-z PMC9335459

[19] Killerby ME, Link-Gelles R, Haight SC, Schrodt CA, England L, Gomes DJ, et al. Characteristics associated with hospitalization among patients with COVID-19—metropolitan Atlanta, Georgia, march–april 2020. Morb Mortal Wkly Rep. 2020;69:790. 10.15585/mmwr.mm6925e1 PMC7316317 32584797

[20] Weatherhead JE, Clark E, Vogel TP, Atmar RL, Kulkarni PA. Inflammatory syndromes associated with SARS-CoV-2 infection: dysregulation of the immune response across the age spectrum. J Clin Invest. 2020;130:6194–7. 10.1172/JCI145301. 33108354 10.1172/JCI145301 PMC7685746

[21] Weiskopf D, Weinberger B, Grubeck-Loebenstein B. The aging of the immune system. Transpl Int. 2009;22:1041–50. 10.1111/j.1432-2277.2009.00927.x. 19624493 10.1111/j.1432-2277.2009.00927.x

[22] Simon AK, Hollander GA, McMichael A. Evolution of the immune system in humans from infancy to old age. Proc R Soc B Biol Sci. 2015. 10.1098/rspb.2014.3085. 10.1098/rspb.2014.3085 PMC4707740 26702035

[23] Minotti C, Tirelli F, Barbieri E, Giaquinto C, Donà D. How is immunosuppressive status affecting children and adults in SARS-CoV-2 infection? A systematic review J Infect. 2020;81:e61–6. 32335173 10.1016/j.jinf.2020.04.026 PMC7179496

[24] Celewicz A, Celewicz M, Michalczyk M, Woźniakowska-gondek P, Krejczy K, Misiek M, et al. Pregnancy as a risk factor of severe COVID-19. J Clin Med. 2021;10:5458. 34830740 10.3390/jcm10225458 PMC8625663

[25] Tian W, Zhang N, Jin R, Feng Y, Wang S, Gao S, et al. Immune suppression in the early stage of COVID-19 disease. Nat Commun. 2020;11:1–8. 33203833 10.1038/s41467-020-19706-9 PMC7673112

[26] McGonagle D, Sharif K, O’Regan A, Bridgewood C. The role of cytokines including interleukin-6 in COVID-19 induced pneumonia and macrophage activation syndrome-like disease. Autoimmun Rev. 2020;19:102537. 32251717 10.1016/j.autrev.2020.102537 PMC7195002

[27] Madrid J, Agarwal P, Müller-Peltzer K, Benning L, Selig M, Diehl P, et al Vaccination protects against mortality and intensive care unit (ICU) admission in hospitalized patients with COVID-19. 2023. Available from: https://www.researchsquare.com.

[28] Levine-Tiefenbrun M, Yelin I, Katz R, Herzel E, Golan Z, Schreiber L, et al. Initial report of decreased SARS-CoV-2 viral load after inoculation with the BNT162b2 vaccine. Nat Med. 2021;27:790–2. 33782619 10.1038/s41591-021-01316-7

[29] Azkur AK, Akdis M, Azkur D, Sokolowska M, van de Veen W, Brüggen MC, et al. Immune response to SARS-CoV-2 and mechanisms of immunopathological changes in COVID-19. Allergy. 2020;75:1564–81. 10.1111/all.14364. 32396996 10.1111/all.14364 PMC7272948

[30] Gallo Marin B, Aghagoli G, Lavine K, Yang L, Siff EJ, Chiang SS, et al. Predictors of COVID-19 severity: a literature review. Rev Med Virol. 2021;31:1–10. 10.1002/rmv.2146. 32845042 10.1002/rmv.2146 PMC7855377

[31] Karami H, Derakhshani A, Ghasemigol M, Fereidouni M, Miri-moghaddam E, Baradaran B, et al. Weighted gene co-expression network analysis combined with machine learning validation to identify key modules and hub genes associated with SARS-CoV-2 infection. J Clin Med. 2021;10:3567. 34441862 10.3390/jcm10163567 PMC8397209

[32] Tang X, Du RH, Wang R, Cao TZ, Guan LL, Yang CQ, et al. Comparison of hospitalized patients with ARDS caused by COVID-19 and H1N1. Chest. 2020;158:195–205. 32224074 10.1016/j.chest.2020.03.032 PMC7151343

[33] Dupont A, Rauch A, Staessens S, Moussa M, Rosa M, Corseaux D, et al. Vascular endothelial damage in the pathogenesis of organ injury in severe COVID-19. Arterioscler Thromb Vasc Biol. 2021;41:1760–73. 10.1161/ATVBAHA.120.315595. 33626910 10.1161/ATVBAHA.120.315595

