# Supplementary material for: Vaccination protects against acute respiratory distress syndrome (ARDS) in hospitalized patients with COVID-19
Source: Clin Exp Med. 2024 Jan 27;24(1):21. doi: 10.1007/s10238-023-01293-w (PMC10822002; doi:10.1007/s10238-023-01293-w)
Supplement: Supplementary file 2 — Primary outcome. Vaccinated versus non-vaccinated hospitalized COVID-19 patients developing ARDS in subgroups adjusted for different confounding factors. Results highlighted in blue are statistically significant Supplementary file2 (DOCX 17 kb) [file 10238_2023_1293_MOESM2_ESM.docx]

Vaccinated and Non-vaccinated Patients developing ARDS

Vaccination Status Without Correction for Confounder Variables With Correction for Confounder Variables

|  | Non-vaccinated | Vaccinated | Risk Ratio (+/- CI) | adj Risk Ratio (+/- CI) |
| --- | --- | --- | --- | --- |
| ARDS (n=167) |  |  | 0.40 (0.21; 0.62) | 0.64 (0.29; 0.94) |
| yes | 62 (37%) 48 (49%) | 14 (20%) |  |  |
| no | 105 (63%) 49 (51%) | 56 (80%) |  |  |
| ARDS (without Omicron n=149) |  |  | 0.46 (0.23; 0.73) | 0.74 (0.34; 0.99) |
| yes | 61 (41%) 48 (51%) | 13 (24%) |  |  |
| no | 88 (59%) 46 (49%) | 42 (76%) |  |  |
| ARDS (without Healthy Subjects n=132) |  |  | 0.41 (0.20; 0.68) | 0.654 (0.27; 0.99) |
| yes | 44 (33%) 31 (47%) | 13 (20%) |  |  |
| no | 88 (67%) 35 (53%) | 53 (80%) |  |  |
| ARDS (without Immunodeficiency n=141) |  |  | 0.37 (0.16; 0.61) | 0.62 (0.23; 0.98) |
| yes | 57 (40%) 47 (52%) | 10 (20%) |  |  |
| no | 84 (60%) 43 (48%) | 41 (80%) |  |  |
| ARDS (without Oncological Disease n=140) |  |  | 0.47 (0.23;0.77) | 0.65 (0.28; 0.99) |
| yes | 59 (42%) 47 (52%) | 12 (24%) |  |  |
| no | 81 (58%) 44 (48%) | 37 (76%) |  |  |
| ARDS (without Vascular Disease n=142) |  |  | 0.46 (0.24; 0.72) | 0.67 (0.31; 0.98) |
| yes | 60 (42%) 47 (53%) | 13 (25%) |  |  |
| no | 82 (58%) 42 (47%) | 40 (75%) |  |  |
| ARDS (without Neurological Disease n=146) |  |  | 0.44 (0.23; 0.72) | 0.63 (0.28; 0.97) |
| yes | 57 (39%) 45 (49%) | 12 (22%) |  |  |
| no | 89 (61%) 46 (51%) | 43 (78%) |  |  |
| ARDS (without Organ Transplant n=152) |  |  | 0.38 (0.19; 0.62) | 0.63 (0.26; 0.97) |
| yes | 59 (39%) 48 (51%) | 11 (19%) |  |  |
| no | 93 (61%) 47 (49%) | 46 (81%) |  |  |
| ARDS (without Pregnancy=161) |  |  | 0.43 (0.22; 0.69) | 0.60 (0.26; 0.95) |
| yes | 56 (35%) 42 (46%) | 14 (20%) |  |  |
| no | 105 (65%) 49 (54%) | 5 (80%)6 |  |  |
